# Supplementary material for: Investigating the influence of physiologically relevant hydrostatic pressure on CHO cell batch culture
Source: Sci Rep. 2021 Jan 8;11:162. doi: 10.1038/s41598-020-80576-8 (PMC7794228; doi:10.1038/s41598-020-80576-8)
Supplement: Supplementary file 1 — Supplementary Information 1. [file 41598_2020_80576_MOESM1_ESM.zip › S1/IgG/Feb17 60 90 standard/standard/80ugml volumn.pdf]

Data File C:\Users\P...s\ChemStation\1\Data\TK_CHO_IgG 2019-02-17 17-28-56\007-6-80ug_ml.D
Sample Name: 80ug/ml

===

Acq. Operator : SYSTEM   Seq. Line : 7
Sample Operator : SYSTEM
Acq. Instrument : HPLC-0039   Location : 6
Injection Date : 2/17/2019 5:56:11 PM   Inj : 1
Inj Volume : 100.000 µl
Different Inj Volume from Sample Entry! Actual Inj Volume : 80.000 µl
Method : C:\Users\Public\Documents\ChemStation\1\Data\TK_CHO_IgG 2019-02-17 17-28-56\CHO_IgG_LC.M (Sequence Method)
Last changed : 2/8/2019 3:17:23 PM by SYSTEM

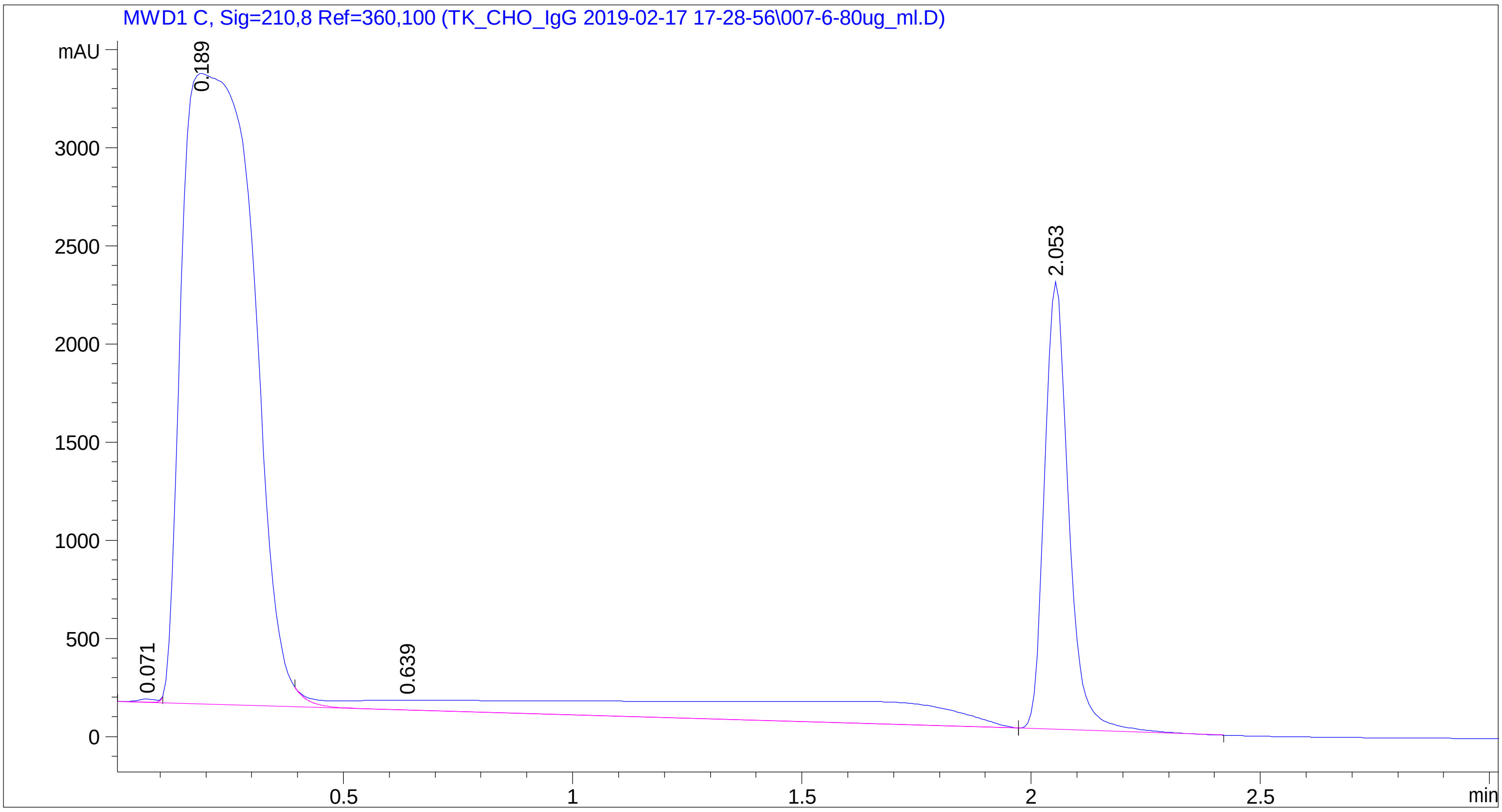

===

Area Percent Report

===

Sorted By : Signal
Multiplier : 1.0000
Dilution : 1.0000
Use Multiplier & Dilution Factor with ISTDs

Signal 1: MWD1 C, Sig=210,8 Ref=360,100

Peak #	RetTime [min]	Type	Width [min]	Area [mAU*s]	Height [mAU]	Area %
1	0.071	BV E	0.0463	47.14988	16.25453	0.0942
2	0.189	VV R	0.1443	3.47517e4	3211.04858	69.4381
3	0.639	VB E	1.6293	6842.24854	49.11614	13.6716
4	2.053	BBA	0.0570	8405.93164	2280.67798	16.7961

Totals : 5.00470e4 5557.09723

===

*** End of Report ***
